# Perspectives on Immunotherapy of Metastatic Colorectal Cancer

**DOI:** 10.3389/fonc.2021.659964

**Published:** 2021-06-09

**Authors:** Yongjiu Dai, Wenhu Zhao, Lei Yue, Xinzheng Dai, Dawei Rong, Fan Wu, Jian Gu, Xiaofeng Qian

**Affiliations:** ^1^ Hepatobiliary/Liver Transplantation Center, First Affiliated Hospital, Nanjing Medical University, Nanjing, China; ^2^ Department of General Surgery, Nanjing First Hospital, Nanjing Medical University, Nanjing, China

**Keywords:** colorectal cancer, liver metastasis, deficient DNA mismatch repair, immunotherapy, immune checkpoint inhibitors, vaccine, adoptive cellular immunotherapy

## Abstract

Colorectal cancer, especially liver metastasis, is still a challenge worldwide. Traditional treatment such as surgery, chemotherapy and radiotherapy have been difficult to be further advanced. We need to develop new treatment methods to further improve the poor prognosis of these patients. The emergence of immunotherapy has brought light to mCRC patients, especially those with dMMR. Based on several large trials, some drugs (pembrolizumab, nivolumab) have been approved by US Food and Drug Administration to treat the patients diagnosed with dMMR tumors. However, immunotherapy has reached a bottleneck for other MSS tumors, with low response rate and poor PFS and OS. Therefore, more clinical trials are underway toward mCRC patients, especially those with MSS. This review is intended to summarize the existing clinical trials to illustrate the development of immunotherapy in mCRC patients, and to provide a new thinking for the direction and experimental design of immunotherapy in the future.

## Introduction

Colorectal cancer (CRC) has received a lot of attention and research due to its high incidence (10.2%) as well as high fatality rate (9.2%) among tumors worldwide (1). Because its early clinical symptoms are atypical and not obvious, CRC is often ignored, leading to delayed diagnosis and treatment. To make the matter worse, approximately 15% of patients had already developed liver metastases at the time of the diagnosis and nearly half of patients progressed to liver metastasis later (2). CRC patients with limited liver metastases lesions may be cured by surgical resection (3). However, a majority of patients are not suitable for surgery due to the following reasons, such as bone or brain metastasis, coexisting systemic diseases, or insufficient residual liver volume (4).

This leads to the need for other novel therapies to improve the poor clinical outcomes of mCRC patients who are not eligible for surgical excision. Chemotherapy, radiotherapy, emerging molecular targeted therapies and combination therapy have demonstrated efficacy for some patients in numerous clinical trials and some of them have been approved for clinical use (5). Among them, the more noteworthy is the emergence of various immunotherapies. Immunotherapies mainly consist of immune checkpoint inhibitors (ICIs), adoptive cellular immunotherapy (ACI) and cancer vaccines. The principle of immunotherapy is to enhance or weaken the function of various immune cells (T cells, NK cells, macrophages, myeloid-derived suppressor cells) to achieve anti-tumor effect (6). These therapies, especially ICIs (anti-PD-1; anti-PD-L1; anti-CTLA-4), have been shown to be effective in patients with CRC that are mismatch repair deficient (dMMR). In other words, immunotherapies including ICIs have a limited effect on those patients with pMMR tumors. More than that, immunotherapy has also been challenged by the increasing discovery of resistance due to mutations and other causes, and the suboptimal stratification of patients by MMR status. This makes immunotherapy combined with chemotherapy and radiotherapy especially molecular targeted therapy get more and more attention and research.

The review aims to expound the rationality and feasibility of the use of immunotherapy in clinical practice by summarizing the existing evidence. Based on an updated analysis of the existing literature, as well as expected results from ongoing and planned clinical trials, we discuss practical strategies for future research targeting novel potential immunotherapies and discuss current barriers.

## Rationale for Immunotherapy in mCRC

### Immune Checkpoint Molecules

**Figure 1:**
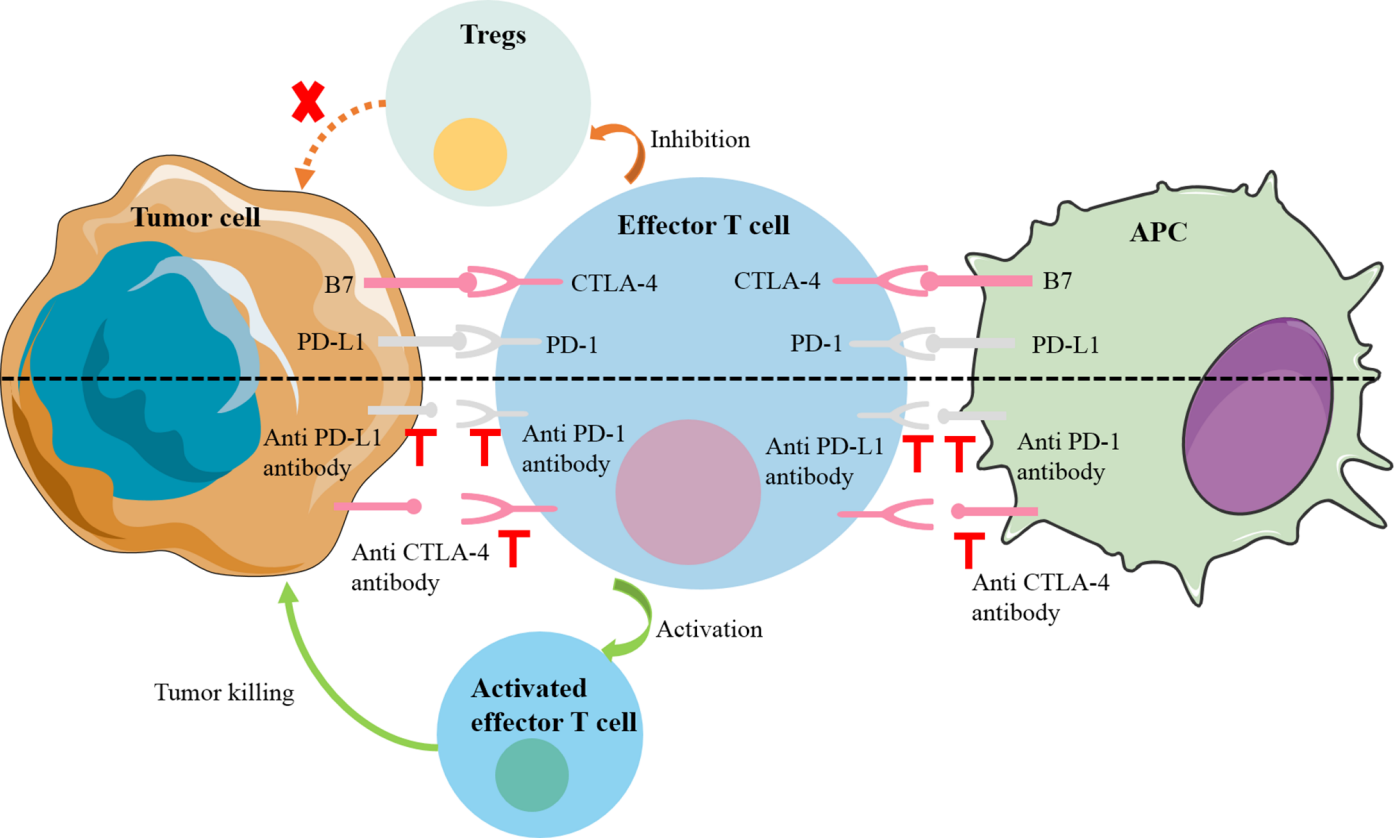
Mechanisms of common immune checkpoint inhibitors. PD1 on the surface of effector T cells interacts with PD-L1 on the surface of tumor cells, downstream signaling pathways are induced to directly inhibit tumor cell apoptosis and stimulate the conversion of effector T cells to Tregs. In a similar manner, CTLA-4 on the surface of T cells can preferentially bind to the receptors (B7-1; B7-2) on the surface of APC to inhibit the activity and proliferation of T cells. APC, antigen-presenting cell; CTLA-4, cytotoxic T-lymphocyte-associated protein 4; PD-1, programmed cell death 1; PD-L1, PD-1 ligand; Treg, regulatory T cell.

Immune checkpoints were originally essential molecules for preventing autoimmunity, but their existence has become a mechanism by which tumors escape the surveillance of the immune system (7). Common immune checkpoint molecules include programmed death cell protein 1 (PD-1), programmed death-ligand 1(PD-L1) and cytotoxic T-lymphocyte-associated protein 4 (CTLA-4). PD-1 is a transmembrane protein, mainly expressed on the surface of a variety of immune cells (e.g., T cells, B cells, dendritic cells, and NK cells) and the corresponding receptor PD-L1 expressed on the surface of tumor cells. The PD-1 signaling pathway can negatively regulate the human immune system, thereby inhibiting the Th1 cytotoxic activity and damaging the host, as did PD-L1 and CTLA-4 (8). Specifically, when PD1 interacts with PD-L1, downstream signaling pathways are induced to directly inhibit tumor cell apoptosis and stimulate the conversion of effector T cells to regulatory T cells (Tregs). In a similar manner, CTLA-4 on the surface of T cells can preferentially bind to the receptors (B7-1; B7-2) on the surface of antigen-presenting cells (APC) due to their higher affinity, so that the activity of T cells is reduced, their proliferation is inhibited, and their anti-tumor effect is weakened (9) (**Figure 1**). These molecules have been found to be overexpressed in solid tumors and in their microenvironment. Wei et al. found that the levels of PD-L1 in liver metastases were higher compared with primary tumors (10). The immune escape of the tumors was reversed by immune checkpoint inhibitors, novel drugs developed to block these negative feedback pathways by binding to PD-1 (nivolumab, pembrolizumab), PD-L1 (atezolizumab), CTLA-4 (ipilimumab). Many existing clinical trials have demonstrated encouraging results in a variety of solid tumors, directly leading to FDA approval of some of these drugs for clinical use (11).

### mCRC With MSI/dMMR or MSS/pMMR

**Figure 2:**
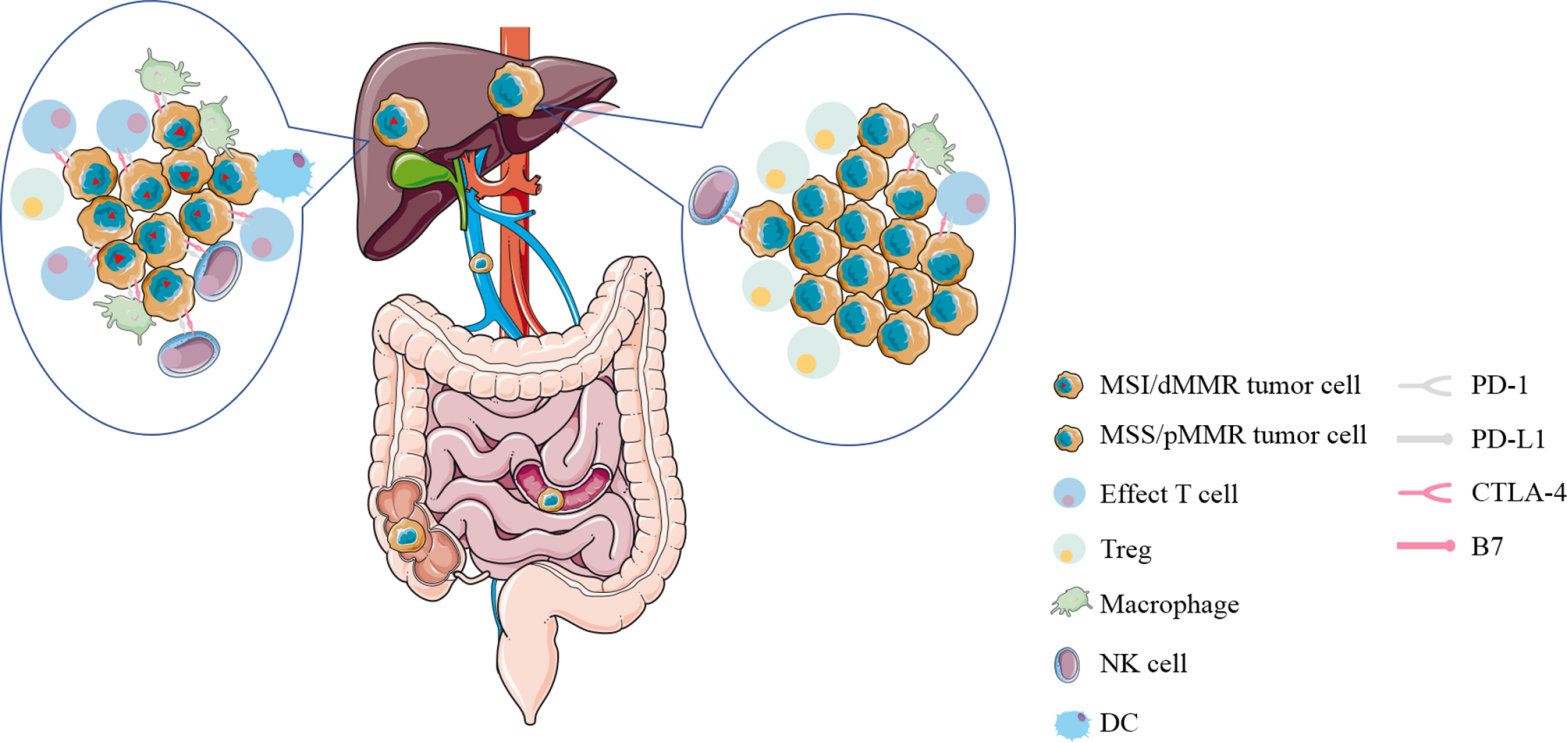
The immune microenvironment of liver metastases from colorectal cancer with MSI/dMMR or MSS/pMMR. Immune cells (CD8+ infiltrating lymphocytes; CD4+ TILS; macrophages; NK cells) are abundant in MSI-H/dMMR tumors and inhibitory checkpoint molecules on the surface of lymphocytes and tumor cells (PD-1, PD-L1, respectively) are increased correspondingly. MSI/dMMR, microsatellite instability/mismatch-repair-deficiency; MSS/pMMR, microsatellite stability/mismatch-repair-proficiency; CTLA-4, cytotoxic T-lymphocyte-associated protein 4; PD-1, programmed cell death 1; PD-L1, PD-1 ligand; Treg, regulatory T cell; DC, dendritic cell; NK cell, natural killer cell.

With the recognition of the potential of immunotherapy to improve some patients with advanced solid tumors, it is apparent that we need new biomarkers that can distinguish between tumors that respond to immunotherapy and those that do not. Some studies showed that there is a strong connection between mutation prevalence and immunotherapy response (12). After that, CRC can be divided into two discrete groups according to the MMR mutation status: MSI/dMMR tumors mainly with high overall mutation burden and MSS/pMMR tumors mostly with relatively much lower mutation burden (13). Sad to say, only about 2–4% of mCRC was diagnosed as MSI/dMMR (14). DNA mismatch repair (MMR) is to ensure the integrity and stability of genetic material by correcting mismatched bases during DNA replication. When the mismatch repair system is defective in the main MMR proteins MLH1, MSH2, MSH6, and PMS2 or microsatellites, multiple mutations accumulate, eventually leading to the development of tumors called mismatch-repair deficiency/microsatellite instability (MSI/dMMR) tumors (15). Immunohistochemistry and PCR are commonly used to diagnose patients with MSI/dMMR or MSS/pMMR. One of the mechanism by which dMMR tumors are sensitive to immunotherapy is the production of multiple neoantigens induced by genomic mutations (16). More importantly, immune cells (CD8+ infiltrating lymphocytes; CD4+ TILS; macrophages; NK cells) are abundant in MSI-H/dMMR tumors and cell surface inhibitory checkpoint molecules of lymphocytes and tumor cells (PD-1, PD-L1, respectively) are increased correspondingly (17, 18) (**Figure 2**). This also means that the corresponding MSS tumor is less likely to respond to immunotherapy, which is showed in multiple studies (19). This is a barrier to immunotherapy that needs to be addressed.

## Immune Checkpoint Inhibitors Therapy

Since mCRC patients’ response to ICIs can vary significantly depending on MMR status, we will focus on mCRC patients with MSI/dMMR or MSS/pMMR here (**Table 1**).

**Table 1:** Summary of Immune Checkpoint Inhibitors Therapies for MCRC.

Study	Phase	Agent	Population	MSI status	Endpoint	Reference
**NCT00730639**	1	anti-PD-1 (MDX-1106)	296 advanced solid tumors, including19 CRC	-	-	(20)
**NCT01876511**	2	anti-PD-1 (pembrolizumab)	41 advanced tumors, including 32 mCRC	dMMR (n = 11) pMMR (n = 21)	The primary endpoints: immune-related objective response rate and the 20-week immune-related progression-free survival rate	(8)
**NCT02060188**	2	anti-PD-1 (nivolumab)	74 recurrent or metastatic CRC	dMMR	The primary endpoints: investigator-assessed ORR	(21)
**NCT02060188**	2	anti-PD-1 (nivolumab) + anti-CTLA4 (ipilimumab)	119 recurrent or metastatic CRC	dMMR	The primary endpoints: investigator-assessed ORR; The secondary endpoints: ORR per blinded independent central review (BICR) and DCR	(22)
**NCT02563002**	3	anti-PD-1 (pembrolizumab) or chemotherapy (5-fluorouracil–based therapy with or without bevacizumab or cetuximab)	307 mCRC	dMMR	The primary endpoints: PFS and OS; The secondary endpoints: OS and safety	(23)
**NCT03150706**	2	anti-PD-L1 (avelumab)	33 mCRC	dMMR (n = 30)	The primary endpoint: ORR	(24)
**NCT02870920**	2	Anti-PD- L1 (durvalumab) + anti-CTLA4 (ipilimumab) + best supportive care (BSC); or BSC alone	180 mCRC	dMMR	The primary endpoint: OS	(25)
**NCT03350126**	2	Anti-PD-1 (nivolumab) plus anti-CTLA4 (ipilimumab)	57 mCRC	dMMR	the frequency of pseudoprogressions (DCR by RECIST and iRECIST at 12 weeks)	(26)
**-**	1	Anti-PD-1 (MDX-1106)	14 advanced mCRC	-	The primary objectives: safety; tolerability; maximum-tolerated dose; pharmacokinetics. The secondary objectives: assessing antitumor activity, pharmacodynamics, immunologic end point	(27)
**-**	2	anti-CTLA4 (tremelimumab)	47 refractory or metastatic CRC	-	The primary endpoints: objective response; The secondary endpoints : safety, duration of response, PFS, and OS	(28)
**NCT02788279**	3	atezolizumab + cobimetinib or atezolizumab monotherapy versus regorafenib	383 advanced or metastatic CRC	MSS	The primary endpoints: OS; The secondary endpoints: investigator-assessed OR, duration of response, and PFS	(19)
**NCT03912857**	2	anti-PD-1(SHR-1210) + apatinib	10 mCRC	MSS	The primary endpoints: ORR; The secondary endpoints: PFS, OS, DCR and safety.	(29)
**NCT02851004**	2	anti-PD-1 (pembrolizumab) + STAT3 inhibitor (napabucasin)	50 mCRC	MSS (n = 40)	The primary endpoints: irORR	(30)
**NCT03406871**	1b	anti-PD-1 (nivolumab) + regorafenib	50 patients, including 25 mCRC	MSS (n = 24)	Secondary objectives: assessing incidences of adverse events, ORR, DCR, PFS, and OS.	(31)

OS, overall survival; PFS, progression-free survival; ORR, objective response rate; irORR, immune-related objective response rate; DCR, disease control rate; BSC, best supportive care; PD-1, programmed cell death 1; PD-L1, programmed cell death ligand-1; CTLA-4, cytotoxic T-lymphocyte–associated protein 4; 5-FU, 5-flourouracil; FOLFOX, 5-flourouracil, leucovorin, oxaliplatin; FOLFIRI, 5-flourouracil, leucovorin, irinotecan; mCRC, metastatic colorectal cancer; MSI, microsatellite instability-(high); MSS, microsatellite stability.

### MSI/dMMR mCRC

The efficacy of ICIs was studied in mCRC patients before the patients were stratified with MSS status. In a phase I study of nivolumab (anti-PD-1) in the 39 patients with treatment-refractory solid tumors, only one mCRC patient (7%, 1/14) achieved a lasting complete response for 6 months (27). Similarly, in another phase I study of nivolumab (n = 296), objective responses were observed only in patients with non-small cell lung cancer, melanoma, or renal cell carcinoma, and not in the mCRC population (0 of 19, 0%) (20). A phase II study of tremelimumab (CTLA4) in the 47 patients with refractory metastatic colorectal cancer also failed, only one patient (2%) achieved partial response (28). These studies suggested that single-agent immune checkpoint therapy is not effective in unselected mCRC. This has led to a shift to research into population-specific immunotherapies in mCRC, such as MSI-H/DMMR mCRC and MSS/PMMR mCRC. In a population-based cohort of 798 mCRC patients, Aasebø et al. reported that the proportion with MSI-H among mCRC patients is nearly twice as high as most previous reports of mCRC (4, 3.5, 4.2, 5%) (21, 22, 32, 33), with 40/583 (7%) tumor samples of MSI-H (23). Wang et al. evaluated the status of MMR and MSI in 40 pairs of situ tumors and liver metastases by immunohistochemistry (IHC) and Polymerase Chain Reaction (PCR) respectively. inconsistent MMR and MSI status were observed in`15% patients (six of 40 patients). There was no significant difference between primary and metastatic tumors in the expression status of MMR (P = 0.1405) (24). Although the proportion of patients with dMMR in metastatic colorectal cancer is not high, the poor prognosis in mCRC patients makes any treatment that can improve survival significant.

Le et al. conducted a phase 2 study that evaluated the clinical efficacy of pembrolizumab(anti-PD1) in the 32 patients with advanced metastatic cancer with and without dMMR. For dMMR mCRC patients, 40% (four of 10 patients; 95% CI, 12 to 74) achieved immune-related objective response and 78% (seven of nine patients; 95% CI, 40 to 97) survive without progression for 20 weeks, compared with 0% (0 of 18 patients; 95% CI, 0 to 20) and 11% (two of 18 patients; 95% CI, 1 to 35) in pMMR CRC. A disease control rate (DCR) of >12 weeks was achieved in 90% dMMR mCRC and 11% pMMR mCRC (8). The efficacy of another anti-PD1 drug nivolumab on MSI/AdMMR mCRC was confirmed in a phase 2 study (CheckMate 142). At a median follow-up of 12.0 months, investigator-assessed objective response (OR) was 31.1% (23 of 74 patients, 95% CI, 20.8–42.9) and DCR for 12 weeks or longer was 69% (51 of 74 patients; 95% CI, 57 to 79%). Two patients (2.7%) had complete responses (CRs) and 22 patients (29.7%) had partial responses (PRs) (25). And on this basis the study further evaluated the role of nivolumab plus ipilimumab(anti-CTLA-4) on MSI/dMMR mCRC patients. At median follow-up of 13.4 months, 55% patients achieved investigator-assessed objective response, and DCR for ≥12 weeks was 80%. Progression-free survival (PFS) rate and overall survival (OS) rate at one year was 71 and 85%, respectively. Surprisingly, in 16 patients (13%) who did not complete the treatment cycle due to immune-mediated toxicity, 63% of these achieved the OR, comparable to the total population (34). A couple of studies above led to FDA approval of pembrolizumab and nivolumab for dMMR CRC previously treated by conventional chemotherapy.

Recently, a phase 3 study comparing the clinical effect of PD-1 blockade and chemotherapy as first-line treatment in MSI-H-DMMR mCRC was reported. Some 307 previously untreated mCRC patients with MSI-H-dMMR were randomly assigned to two groups at a ratio of 1:1, and received 200 mg of pembrolizumab every 3 weeks or chemotherapy every 2 weeks, respectively. At a median follow-up of 32.4 months, median PFS was 16.5 months for the pembrolizumab group, compared with 8.2 months for the chemotherapy group, respectively (P = 0.0002). About 43.8% patients in the pembrolizumab group had OR and 33.1% in the chemotherapy group. In addition, 22% of patients in the pembrolizumab group experienced treatment-related adverse events of grade 3 or higher, while 66% in the chemotherapy group (including one death). As a first-line treatment for MSI-H-DMMR metastatic colorectal cancer, pembrolizumab can remarkably improve PFS and reduced treatment-related adverse events, compared with chemotherapy (35). This further suggests that for MSI-H-DMMR metastatic colorectal cancer, chemotherapy is not recommended and immunotherapy should be accepted. But the remarkable thing is that 153 (50%) had synchronous liver metastases, and 77 (25%) had BRAFV600E mutant tumors. This means that more accurate stratification is needed to further study the efficacy of immunotherapy and chemotherapy on MSI-H-DMMR mCRC patients.

Additionally, the anti-PD-L1 therapy of patients has also been increasingly studied. In a phase II study of Avelumab in the 21 patients with dMMR/MSI-H mCRC, complete response rate (CRR) and partial response (PRR) were both 14.3% (three patients), with ORR and DCR of 28.6 and 90.5%, respectively. At a median follow-up of 16.3 months, median PFS was 8.1 months (95% CI, 1.1 to 15.1 months) (36). Chen et al. conducted a phase 2 study to assess whether combination therapy with anti-PD-L1 and anti-CTLA-4 is effective in patients with intractable mCRC. With a median follow-up of 15.2 months, the median OS was 6.6 months for durvalumab and tremelimumab, compared with 4.1 months for best supportive care (BSC) alone (P = .07). However, PFS was 1.8 and 1.9 months respectively. There was no CR. It is worth noting that durvalumab plus tremelimumab significantly improved OS in MSS patients (HR, 0.66; 90% CI, 0.49–0.89; P = .02). This underlines the possibility of combining immunotherapy in unselected advanced mCRC (37).

What is the effect of immunotherapy as neoadjuvant therapy in perioperative period? In a retrospective study of eight patients with advanced MSI-H CRC, pathologic complete response was observed in five of the seven resected patients, and clinical complete response was observed in an unoperated patient (26). In another retrospective analysis of 121 advanced dMMR mCRC patients treated with ICIs, 13 patients achieved pathologic complete response as is shown in the resected specimens. Preoperative imaging in 12 of those patients, however, still showed residual tumor. The result indicates that patients with residual radiographic tumors may not need surgery based on anti-PD1 response (38). In general, the possibilities of ICIs for mCRC continue to expand.

Although ICIs has made significant progress in dMMR mCRC, the objective response rates shown in various studies were still unsatisfactory. This may be due to the following reasons. The first reason is misdiagnosis. In a *post hoc* analysis of 38 patients with mCRC diagnosed as MSI/dMMR, five individuals (13%) were resistant to immune checkpoint inhibitors. After reassessment of the status, three of these patients (60%) were confirmed as MSS/pMMR. Misdiagnosis of their MSI/dMMR status is the main cause of the resistance to ICIs in mCRC shown as MSI/dMMR. Therefore, Cohen et al. advocated that immunohistochemistry and polymerase chain reaction should be combined routinely to detect of MSI/MMR status prior to ICIs. But this increases the cost of the tests, which may be bad for future adoption (39). The second reason is tumor heterogeneity. Although MSI is considered to be an early event of CRC, there is the possibility of heterogeneity in MSI/dMMR tumors. In a case report, a mCRC patient was found to possess immunohistochemical and molecular heterogeneity in MSI/dMMR status in the primary tumor. Significantly, treatment with nivolumab plus ipilimumab showed clear clinical benefit for the patients, with a deep and lasting response. This conclusion needs to be further confirmed by large sample studies (40). The presence of pseudoprogression (PSPD) is also a possible cause. After treatment, the phenomenon of enlargement of the original lesion or the appearance of new lesion, which is similar to the recurrence of tumor, called pseudoprogression. Pseudoprogression could be misjudged as unresponsive status, resulting in the difficulties with the following treatment choices. Colle et al. retrospectively analysis the data of 123 patients with MSI/dMMR mCRC treated with ICIs. About 10% of the population (12/123) experienced PSPD. The median time to PSPD was 5.7 weeks (95% CI, 4.1–11.4), however, after 3 months, no one experienced PSPD. Some nine of 61 patients (14.8%) had PSPD in the anti-PD1 alone group, compared with three of 62 patients (4.8%) in the anti-PD1 plus anti-CTLA-4 group. These results suggest that iRECIST criteria should be questioned after 3 months of immunotherapy (41). In a phase II study of 57 patients with MSI/dMMR mCRC treated with nivolumab and ipilimumab, only 3.5% (2/57) patients experienced PSPD. This result is consistent with the previous study (42). Parseghian et al. found that PSPD was not seen in 59 MSS mCRC treated with immunotherapy, which may be related to its poor efficacy (29).There are many other mechanisms of drug resistance in tumors. It is well known that CD8+ cytotoxic T lymphocytes (CTLs) are the main immune cells that kill target tumor cells in cancer immune surveillance. In terms of mechanism, Fas-FasL apoptosis pathway plays an important part (43). Fas is a cell surface receptor of the tumor necrosis factor receptor superfamily, which is expressed multiple kinds of cells including tumor cells. FasL is also a member of the tumor necrosis factor superfamily, but it is selectively expressed in activated T cells and NK cells. The binding of Fas and its ligand FasL induces the trimerization of Fas receptors, and then forms the death-inducing signal complex (DISC) in the cytoplasmic region of Fas receptors, and cleaves procaspase-8 at DISC, resulting in Fas-mediated cells apoptosis (44). *In vivo* and *in vitro*, Xiao et al. found that Fas expression was decreased in a subset of CD133+CD24lo colon cancer cells, leading to immune evade (31). In addition, the neoantigen is likely to form a complex with human leukocyte antigen class I (HLA class I) on the surface of tumor cells and presented by antigen-presenting cells in dMMR tumors. However, it has been reported that HLA class I expression defects occur in most dMMR CRC, which will prevent the antigen presentation of these tumors (30, 45). Likewise, Ijsselsteijn et al. determined that majority (73–78%) of dMMR cases in two independent cohorts of CRC had loss of HLA class I expression, which may cause immune escape (46).

### MSS/pMMR mCRC

Unfortunately, MSS/pMMR mCRC patients in the ICIs trials failed to gain any clinically significant response or survival benefit from either monotherapy or dual therapy. Eng et al. reported a phase III study of 363 patients with MSS mCRC treated with atezolizumab plus cobimetinib or atezolizumab monotherapy or regorafenib in the third-line setting.After a median follow-up of 7.3 months, Median overall survival was 8.87 months (95% CI 7.00–10.61) in the atezolizumab plus cobimetinib group, 7.10 months (6.05–10.05) in the atezolizumab group, and 8.51 months (6.41–10.71) in the regorafenib group. None of the three groups achieved complete response. Partial response rate was 3% (five of 183) in the combination group, 2% (two of 90) in the atezolizumab group, 2% (two of 90) in the regorafenib group. In general, there is no significant difference across all three groups in OS, PFS, OR, and duration of response (19). In a phase II study of 10 patients with MSS mCRC treated with SHR-1210 (anti-PD-1) plus apatinib, no one (0%) achieved OR and two (22.2%) patients achieved disease control. The median PFS and the median OS was 1.83 months (95% CI, 1.80–1.86 months) and 7.80 months (95% CI, 0–17.07). In conclusion, MSS mCRC failed to benefit from SHR-1210 combined with apatinib (47). In a retrospective study of 23 MSS or pMMR mCRC treated with regorafenib plus antiPD-1 antibody, ORR was 0% and DCR was 78.3% (18/23), with the median PFS of 3.1 months (95% CI, 2.32–3.89). The results are consistent with clinical trials above (48). In another retrospective study, Wang et al. found that MSS CRC patients with no history of liver metastasis are more likely to benefit from this combination regorafenib plus antiPD-1 antibody (49).

However, the recent results of a phase Ib trial suggest the encouraging antitumor activity of regorafenib plus nivolumab in MSS mCRC, with ORR of 36% (9/25) and median PFS of 7.9 months in mCRC (50). In a recent phase II clinical trial, mCRC patients with MSS seemed to benefit from napabucasin (STAT3 inhibitor) plus pembrolizumab, with irORR of 10.0% (four of 40 patients; 95% CI, 2.8–23.7) (51). These conflicting results indicate that the combination of molecular targeted therapy with immunotherapy remains controversial for MSS patients and there is no conclusive evidence to validate its efficacy. With clinical trials of multiple molecular targeted therapies under way, this remains a promising therapeutic strategy for MSS patients. Before the era of immunotherapy, the efficacy of chemotherapy alone in CRC patients with MSS is also limited. In an ACCENT pooled analysis of seven studies, survival time after recurrence in stage III CRC patients with MSS/pMMR treated with adjuvant chemotherapy was shorter compared with those MSI/dMMR patients (52). Martin-Romano et al. reported that pts with refractory MSS mCRC might benefit from chemotherapy after ICI. In the retrospective study of 29 pts with mCRC received chemotherapy after ICI failure [MSS tumors, 27 pts (86%)], four patients (19%) achieved partial response and 9 pts (43%) achieved stable disease, with disease control rate of 62%. The median PFS and OS were 3.8 months (95% CI = 1.5–5.4) and 8.0 months (95% CI = 4.2–14.0), respectively. Since single chemotherapy or single immunotherapy is not effective, this also suggests the potential efficacy of chemotherapy combined with immunotherapy in MSS patients (53).

### Biomarkers of Immune Response

Microsatellite instability (MSI) is recognized as a biomarker to predict the response to ICIs in solid tumors. The KEYNOTE-016 trial underlined the utility of MSI-H:dMMR as a predictive biomarker to antiPD-1 therapy (pembrolizumab) in mCRC (8). High DCR and beneficial PFS were observed in in mCRC patients with MSI-H treated by PD-1 inhibitors; however, less than half of the patients had clinical response, suggesting that patients needed additional predictive biomarkers. MSI-H tumors tend to have high tumor mutational burden (TMB), and studies have demonstrated that TMB is commonly increased in MSI-H mCRC, but still unclear (54). In mCRC patients treated with durvalumab and tremelimumab, OS is the greatest in MSS Patients with more plasma TMB of 28 variants per megabase or more (HR, 0.34; 90% CI, 0.18-0.63; P = .004) (37). Schrock et al. analyzed TMB in 22 patients treated with PD-1/L1 inhibitors, TMB was strongly associated with objective response (OR; P <0.001) and PFS, by univariate (P <0.001) and multivariate analysis (P <0.01). At a median follow up of 18 months, patients with high TMB has not reached the median PFS while patients with low TMB had median PFS of only 2 months. In MSI-H mCRC, TMB appears to be a crucial independent biomarker, which can stratify patients who may respond to ICIs (55). By analyzing CRC tissue sections, 164 of 5,702 (2.9%) MSS cases were assessed as TMB-high. It means that more people may benefit from ICI When TMB was used as a prognostic marker (56). However, based on the clinical response data collected from six patients with metastatic MSIH/DMMR GI cancers treated by ICIS, Hirsch et al. found that TMB wasn’t associated with extent and duration of response (57). By comparing the expression of 44 selected immune-related genes in the primary colon tumor between responders (n = 13) and nonresponders (n = 6) after anti-PD-1 therapy, Llosa et al. concluded that preexisting antitumor immune response has little predictive value for immunoreactive pMMR CRC (58). A growing body of evidence suggests that infiltrating lymphocytes are inextricably associated with TMB, infiltrating lymphocytes is also an important prognostic marker for CRC patients after ICIs. High infiltrating lymphocytes densities (CD3, CD8, FoxP3, and CD45RO) had significant correlation with improved overall survival for primary colorectal cancer (all p <0.001). Moreover, the densities of CD8 cells predicted the good tumor regression grade well in locally advanced rectal cancer after chemoradiotherapy (59). In a study, Loupakis et al. collected data from 85 patients with MSI-H mCRC treated with ICIs. RR in patients with high number of TILs (TILs-H) and those with low number of TILs was 70.6 and 42.9%, respectively (odds ratio = 3.20, p = .0291). Patients with TILs-H had better survival outcomes than those with TILs-L (PFS: not reached *vs* 27.8 months, HR = 0.42, p = .0278; OS: HR = 0.41, p = .0463) (60).

In addition to these now routinely studied, some new biomarkers are increasingly being studied. The levels of B7-H3, B7-H4, and PD-L1 protein in tissues from 805 primary tumors and matched metastases were evaluated by microarrays. Detectable rate of B7-H3, B7-H4 and PD-L were 50.9, 29.1 and 29.2%, and elevated B7-H3 expression had an association with advanced overall stage. B7-H3 overexpression in primary tumors predicted poor DFS, while B7-H4 and PD-L1 had no significant relationships with survival. Overall, B7-H3 had a higher expression rate than B7-H4 and PD-L1, and was significantly associated with poor prognosis (61). Lu et al. found mCRC patients with early decrease in serum interleukin 1 receptor antagonist had longer PFS (not reached *vs* 2.1 months; HR = 0.06; 95% CI, 0.01 to 0.38; P <.001). Compared with MSI status or PD-L1 expression, an early decrease in serum interleukin 1 receptor antagonist can better determine who will respond to ICIs in patients with metastatic CRC (62). A case was reported that a mCRC patient who carried the rare 9p24.1 CNG achieved a lasting partial response after immunotherapy, which may support the use of ICIs in solid tumors carrying the rare 9p24.1 CNG (63). The evaluation of TMB and TILs should be incorporated into future trials of ICIs in mCRC to confirm our results and to explore methods and threshold issues for routine clinical use.

## Adoptive Cellular Immunotherapy

Generally, autologous T cells were targeted to tumor specific antigens by gene editing, then were injected back into the patient to stimulate the host antitumor immune response. As significant efficacy was reported in a large amount of hematologic malignancies and solid tumors, adoptive T-cell therapy is recently another novel immunotherapy option for mCRC patients. In gastrointestinal tumors, cancer embryonic antigen (CEA) is a sensitive tumor biomarker, which can be detected in CRC tissues and serum with increased levels. In one of the earliest clinical trials, three refractory mCRC patients were administered autologous T lymphocytes genetically engineered to express a murine T cell receptor (TCR) against human carcinoembryonic antigen (CEA). Levels of CEA in serum were profoundly decreased in all patients (74–99%), and objective shrinkage of liver and lung metastatic lesions was observed in one patient, although a severe transient inflammatory colitis was observed in all three patients (64). In a phase I study of CEA CAR-T cell in 10 CEA+ mCRC patients, seven progressive patients had stable disease after CAR-T therapy. Among them, two patients maintained more than 30 weeks, and two patients showed tumor regression. In conclusion, most treated patients achieved some efficacy (65). Here Hege et al. report results of trials of CAR-T cells targeting tumor-associated glycoprotein (TAG)-72 (CART72 cells) in the treatment of metastatic colorectal cancer. CART72 cells in blood last for a short time (≤14 weeks), and CART72 cells in tumor tissues can be detected in tumor biopsy from one of three patients. CART72 cells had limited efficacy in mCRC, suggesting that incorporation of co-stimulatory domains in the CAR design was needed (66). The study showed that postoperative CRC patients may benefit from adjuvant sentinel lymph nodes lymphocyte (SLN-T) immunotherapy. 1-year survival rate in SLN-T lymphocyte group was 55.6%, compared with 17.5% in the control group (p = 0.02). The median OS of the SLN-T lymphocyte was 28 months, compared with 14 months of the control group (67). In addition, specific T cells targeting other neoantigens detected in tumor tissue can also be used for treatment. In a case report, objective regression of all seven lung metastases was observed after the transfusion of tumor-infiltrating lymphocytes specifically targeted KRAS G12D, which was identified in tumor-infiltrating lymphocytes obtained from a patient with metastatic colorectal cancer (68). In another case report, tumor-infiltrating lymphocytes with HLA-A*0201-restricted recognition of mutated p53 p.R175H were identified, which can mediated recognition of multiple epithelial cancers that expresses both HLA-A*0201 and the p53 p.R175H mutation (69). CD4+ and CD8+ memory T cells targeting the mutated KRASG12D and KRASG12V variants respectively in the peripheral blood of cancer patients were conformed and isolated, suggesting that we can detect memory T cells targeting distinct or common somatic mutations in the peripheral blood of epithelial cancer patients and can hopefully use them to develop efficient individualized T cell-based cancer immunotherapy among a variety of patients (70).

Because NK cells can induce antitumor activity, independent of antigen and major histocompatibility complex (MHC), increasing clinical trials are testing the efficacy of adoptive cancer therapy with NK cells. NK cells treatment effectively extend the lives of leukemia patients. Due to good therapeutic effect and safety, NK cell therapy is considered to be superior to adoptive therapy of autologous T cells, however, many clinical trials of NK cells in solid tumors failed to achieve end points. Veluchamy et al. confirmed the antitumor efficacy *in vivo* and *in vitro* where umbilical cord blood stem cell-derived NK cells (UCB-NK) showed enhanced antitumor cytotoxicity against colon cancer cells independent of EGFR and RAS status (71). Xiao et al. used CAR-NK cells fusing the extracellular domain of the natural killer (NK) cell receptor NKG2D to DAP12 to treat three mCRC patients. Ascites and number of tumor cells in ascites samples were decreased in the first two patients after treatment with intraperitoneal injection of the CAR-NK cells. The third patient with liver metastatic experienced tumor regression in the liver region after treatment with intraperitoneal infusion of the CAR-NK cells following percutaneous injection (72).

In addition, the synergistic anti-tumor immunity of T cells and NK cells can also be achieved by targeting NKG2D for T cells. In an animal experiment, tumor burden was significantly reduced in established peritoneal colorectal xenografts after treatment with CAR-T cells specific for NKG2D ligands (73).

## Vaccine Therapy

Colorectal cancer overexpressed some common tumor associated antigens, which can serve as a target for vaccine in immunotherapy. Multiple types of vaccines studied in mCRC include autologous, peptide, and dendritic cell vaccines (**Table 2**). A phase I/II trial of p53 synthetic long peptide (p53-SLP) vaccine was performed in ten mCRC patients. p53-specific T-cell reactivity (≥6 months) was observed in 67% patients (six of nine), however, polarized p53-specific CD4 + T cells accounted for only a small proportion. How to improve the polarization of the p53-SLP vaccine-induced T-cell response should be focused in future trials (75). Balint et al. observed the decreased Treg to Teff cell ratio in samples from three of five patients and increased cytolytic T cell responses after immunizations in a phase 1/2 clinical trial of advanced-generation Ad5 [E1-, E2b-]-CEA(6D) vaccine in mCRC patients. After a long-term follow-up, 20% of patients were still alive, with median survival of 11 months (79). Morse et al. demonstrated that patients produced less neutralizing antibodies and more CEA-specific T cell responses when using VRP as vectors in a phase I/II study. In a further study, the 5-year RFS was 75% in patients with stage III cancer (95%CI 40 to 91%) and no one died. CD8+T_EM_ increased and FOXP3 + Tregs decreased in 83% patients (10/12) after vaccination treatment. The results suggested that VRP-CEA may prolong the OS in stage III CRC patients (76, 80). A Randomized Clinical Trial reported that PFS and OS of mCRC patients in the modified vaccinia Ankara-5T4 (MVA-5T4) treatment group was significantly prolonged, compared with those in the no treatment group (5.6 months *vs* 2.4 months,P <.001; 20.0 *vs* 10.3 months; HR, 0.32; 95% CI, 0.14–0.74; P = .008).In addition, baseline anti-5T4 responses was doubled in 16 of 35 mCRC patients treated with MVA-5T4 (81). It is worth mentioning that the vaccine with poxvirus vectors highlights a critical component of vaccine therapy. Poxvirus vectors can be used to incorporate multiple transgenes and its safety has been increasingly proven. In a pilot study of 25 patients treated with a poxviral vaccine regimen targeting CEA and MUC-1, along with a triad of costimulatory molecules engineered into vaccinia (PANVAC-V) as a prime vaccination and into fowlpox (PANVAC-F) as a booster vaccination, the vaccine was tolerable and nine of 16 patients achieved immune responses to MUC-1/CEA (77). A randomized phase II study further study the therapeutic effect of vaccines based on dendritic cells (DCs) and poxvectors targeting CEA and MUC1 (PANVAC) in resected mCRC patients. Patients (n = 74) were randomized to injections of autologous DCs modified with PANVAC (DC/PANVAC) or PANVAC with per injection GM-CSF. Two-year recurrence-free survival in DC/PANVAC and PANVAC/GM-CSF group was 47 and 55% respectively (P = 0.48). In addition, the vaccinated patients have better survival than the unvaccinated group (78). An open-label, 3 + 3 design, dose-escalation trial proved the safety and potential clinical activity of a new poxviral-based vaccine (BN-CV301), comprised of recombinant (rec.) modified vaccinia Ankara (MVA-BN-CV301; prime) and rec. fowlpox (FPV-CV301; boost) (74).

**Table 2:** Summary of Vaccine Treatment for mCRC.

Study	Phase	Agent	Population	MSI status	Endpoint	Reference
**NCT01147965**	1/2	AD5-CEA Vaccine	32 mCRC	-	The primary purpose: determine the safety The secondary objectives: evaluate CEA-specific immune responses and clinical response rate	(68)
**NCT00529984**	2	AVX701 (VRP-CEA Vaccine)	28 metastatic tumors; including 21 mCRC	-	the primary objectives: determine the safety The secondary objectives: evaluate CEA-specific immune responses and clinical response rate	(69)
**NCT01890213**	2	AVX701 (VRP-CEA Vaccine)	12 Stage III CRC	-	-	(70)
**NCT00154713**	1	CEA-pulsed DC	12 mCRC	-	The primary endpoint: safety	(74)
**NCT01462513**	2	Tecemotide (L-BLP25) or placebo	121 mCRC with R0/R1 resection		The primary endpoints: RFS and 3-year overall survival (OS) rate; The secondary endpoints: RFS and OS in subgroups with different MUC1 expression and safety	(72)
**NCT01461148**	1/2a	FSP-based vaccine	22 CRC	MSI	The primary endpoints: safety (phase I) and immunogenicity (phase IIa); The secondary endpoints: tumor response (both phases) and immunogenicity (phase I) and safety (phase IIa)	(73)
**NCT00027833**	2	ALVAC-CEA-B7.1 vaccine + FOLFIRI; FOLFIRI + ALVAC-CEA-B7.1 vaccine; ALVAC-CEA-B7.1 vaccine + tetanus toxoid + FOLFIRI	180 mCRC	-	The primary endpoints: Immune response to the vaccine.	(75)
**NCT00676949**	1	5 peptide vaccines of KOC1, TTK, CO16, DEPDC1, MPHOSPH1	18 metastatic Tumors, including nine mCRC	-	The primary end point: safety and tolerability. The secondary endpoints: MTD and immune response	(76)
**NCT01413295**	2	DC vaccine + BSC or BSC alone	52 mCRC	-	The primary endpoints: PFS; The secondary endpoints: PFS, OS, toxic effects, and ORR.	(77)
**NCT01348256**	2	DC vaccine	19 mCRC	-	-	(78)
**-**	1/2	p53-SLP	10 mCRC	-	-	(67)
**-**		MVA-5T4, metronomic low-dose cyclophosphamide, or a combination of both treatments	55 mCRC	-	The primary endpoints: magnitude of 5T4-specific responses at treatment day 43; The secondary end points: the kinetics of anti-5T4 immune responses overtime, PFS, OS	(71)
**-**	2	TroVax(MVA-5T4)	19 mCRC	-	-	(79)
**-**	2	a peptide vaccine combined with UFT/LV	46 stage III CRC	-	The primary end point: RFS; The secondary endpoints: OS, safety, tolerability and peptide-specific activities	(80)
**-**		DC vaccine	46 mCRC	-	-	(81)

OS, overall survival; PFS, progression-free survival; RFS, recurrence-free survival; ORR, objective response rate; irORR, immune-related objective response rate; DCR, disease control rate; BSC, best supportive care; FOLFIRI, 5-flourouracil, leucovorin, irinotecan; CEA, carcinoembryonic antigen; FSP, frameshift peptide; DC, dendritic cell; mCRC, metastatic colorectal cancer; MSI-(H), microsatellite instability-(high); MSS, microsatellite stable.

There are also new tumor-associated antigens being developed for use in vaccine therapy. A phase 2 study was performed to test the efficacy of tecemotide (an antigen-specific cancer vaccine inducing immunity against mucin-1). There is no significant difference in RFS and 3-year OS rate between mCRC patients after resection of CRLM treated with tecemotide and those treated with placebo (82). Accumulated abundant insertion/deletion mutations in dMMR cancer cells at microsatellites resulted in the production of immunogenic frameshift peptide (FSP) neoantigens. Kloor et al. performed a clinical phase I/IIa trial of FSP-based vaccine in dMMR CRC. All patients achieved humoral and cellular immune responses induced by the vaccine. However, only two patients (9%, two of 22 patients) achieved stable disease as best overall response. Among them, stable disease and stable CEA levels (≥7 months) was observed in a severely metastatic patient received extensive treatment (83).

What is the effect of a combination of vaccine and chemotherapy? Kaufman et al. conducted a study to assess whether systemic chemotherapy can affect the on T-cell immunity induced by ALVAC-CEA/B7.1 vaccine in mCRC patients. The vaccine was injected before and after treatment with 5-fluorouracil, leukovorin and irinotecan. The generation of CEA-specific T-cell responses following vaccination was not affected by systemic chemotherapy, with no differences in clinical or immune response across the treatment groups (84). Similar results were found in another study of MVA-5T4 (TroVax) and systemic chemotherapy in 19 mCRC patients (85). The benefits of the vaccine combined with chemotherapy for patients have been further confirmed in subsequent studies. HLA-A2402+ patients with advanced solid tumors (nine colorectal cancer) were treated with vaccine composed of five HLA-A2402-restricted, tumor-associated antigen (TAA) epitope peptides, following by escalating doses of cyclophosphamide. After treatment of cyclophosphamide, regulatory T cells baseline was decreased. TAA-specific T cell responses were significantly collected to longer overall survival (86). A similar phase II clinical trial of a peptide vaccine combined with UFT/LV as adjuvant treatment was performed in patients with stage III CRC. Three-year RFS rate was 85.7% in patients with positive CTL responses in the HLA-A*2402 matched group, compared with 33.3% in those without (HR = 0.159, 95% CI: 0.023–0.697; P = 0.011), although there was no significant difference in three-year RFS between HLA-A*2402 matched and unmatched groups (67.8 *vs*. 73.6%, respectively; HR = 1.254, 95% CI: 0.48–4.63; P = 0.706) (87).

Because dendritic cell cells (DCs) are the most effective antigen-presenting cells, it is possible to exploit their diversity to produce improved therapeutic vaccines. Many therapeutic vaccination routes against cancer are being developed clinically. Twenty-six colorectal patients received DCs treatment after resection of the metastatic lesion. 5-year RFS rate was 63% in patients with evidence of a vaccine-induced immune response 1 week after vaccination, compared with 18% in nonresponders (P = 0.037) (88). In another phase II trial, pre-treated mCRC patients were randomly assigned to receive autologous tumor lysate dendritic cell vaccine (ADC) + best supportive care (BSC) (experimental arm [EA]) or BSC (control arm [CA] alone. No one in EA achieved objective radiological response. Median PFS was 2.7 months (95% CI, 2.3–3.2 months) and 2.3 months (95% CI, 2.1–2.5 months) (p = 0.628),median OS was 6.2 months (95% CI, 4.4–7.9 months) and 4.7 months (95% CI, 2.3–7 months) in the CA *vs*. EA group (p = 0.41), respectively. OS in responders was 7.3 months (95% CI, 5.2–9.4 months), compared with 3.8 months (95% CI, 0.6–6.9 months) in non-responders (p = 0.026).The results mean that patients don’t benefit from ADC, although ADC-induced tumor-specific immune response was observed in patients (89). However, Rodriguez et al. reported that mCRC patients who received the DC vaccine as postoperative adjuvant therapy were less likely to relapse, with median DFS of 25.26 months in the vaccine arm versus 9.53 months of months in the observation arm (90). Dendritic cell vaccines that target specific tumor-associated antigens may further enhance the effectiveness of immunotherapy. In twelve patients treated with CEA-pulsed DCs mixed with tetanus toxoid and subsequent interleukin-2, two patients had stable disease and 10 patients showed disease progression, suggesting that a small proportion of patients had clinical benefit (91). In another phase I study of DC vaccination targeting WT1 for resectable advanced CRC patients, patients achieved lasting immunity from DC vaccination (≥2 years) and prolonged survival (92).

In addition to developing a variety of vaccines, the corresponding vector is also under continuous research to better enhance the immune response. Adenovirus serotype 5 (Ad5), as a common viral vector, is often used to prepare vaccines against pathogens and tumor antigens. However, AD5-induced virus-specific neutralizing antibodies appear after exposure to an AD5-based vaccine, limiting transgenic transmission and target-specific immunity. Flickinger et al. reported that more patients (≥90%) with Ad5.F35-based vaccines targeting tumor antigens achieved clinically relevant immune responses, compared with approximately 50% patients with Ad5-based vaccines (93).

Attempts to confirm the efficacy of the vaccine for mCRC through multiple ways (e.g., dendritic cells, autologous tumor cells, recombinant viral vectors, and peptides) appear to have failed, with limited clinical efficacy and outcomes, despite improved specific immune responses.

### The Combination of Immunotherapy and Targeted Therapy

In recent years, a variety of targeted therapies led by anti-angiogenic drugs have been increasingly used in the clinical treatment of various tumors. In addition to their excellent anti-angiogenic effects, they also have immune-enhancing effects. Manzoni et al. found that patients responded to bevacizumab showed a trend of increasing CD3 (p = 0.07) and CD4 (p = 0.05) (94). By analyzing immune cell infiltration in the liver metastatic sites of 53 colorectal cancer patients treated with chemotherapy plus cetuximab, chemotherapy without cetuximab, and no chemotherapy before operation, Inoue et al. reported that the chemotherapy with cetuximab group had a higher infiltration of CD3+ (P = 0.003), CD8+ (P = 0.003) and CD56+ (P = 0.001) cells, compared with other groups (95). This opens up new possibilities to further improve clinical outcomes in combination with immunotherapy, especially for immunotreatment-resistant MSS tumors. A single arm, multi-center phase II study (CAVE Colon) was conducted to study the efficacy of avelumab and cetuximab in RAS WT mCRC patients treated with a first-line CT in combination with an anti-EGFR agent with a major response achieved (complete or partial). We are looking forward to its clinical trial results (96). In another single arm phase II AVETUX trial, 43 RAS/BRAF wildtype mCRC pts (40 MSS) received the treatment of mFOLFOX6 and cetuximab combined with avelumab. The ORR and DFS were 79.5 and 92.3% respectively. Among them, 6 pts had CR and 25 pts had PR.; 2 pts had progression and 1 was not evaluable. In addition, 79.5% patients achieved early tumor shrinkage (ETS) (≥20% after 8 weeks). In short, The AVETUX regimen is feasible and produces a high response rate in MSS patients, mainly occurring in the first 8 weeks (97). However, 445 BRAFwt mCRC pts in MODUL study who received 16 weeks of induction treatment with FOLFOX + BEV were randomized to take medication of FP/BEV + atezolizumab (297 pts) or FP/BEV (148 pts). At a median follow-up of 10.5/18.7 months there was no significant difference in PFS and OS. Adding atezolizumab to FP/BEV as a first-line treatment did not benefit BRAFwt mCRC patients (98). Recently, MEK inhibitors have received increasing attention, particularly in the area of combined immunotherapy, whose efficacy has been evaluated in multiple clinical trials. In a phase I/Ib study of MEK inhibitor (cobimetinib) and PD-L1 inhibitor (atezolizumab) in patients with solid tumors (mCRC; n = 84), 8% mCRC patients (seven of 84 patients) achieved confirmed responses, independent of KRAS/BRAF status across diseases. However, potential collaborative activity observed in mCRC disappeared in a further phase III study (99). Due to the potential to induce antibody-dependent cell-mediated cytotoxicity (ADCC), stimulation of NK cells represents another ideal target for this molecular approach. In LOVO xenograft tumor models with positive EGFR expression, the combination of cetuximab and NK cells showed great antitumor effect (100). Similarly, cetuximab enhanced the cytotoxic activity of NK cells on EGFR+ tumor cells independent of RAS status (101).

### The Combination of Immunotherapy and Chemotherapy

Tumor associated antigens, such as CEA and other specific molecules, tend to be overexpressed as chemotherapeutic drugs kill tumor cells. Meanwhile, death signaling induced by tumor antigen-specific cytotoxic T lymphocytes during chemotherapy moderates tumor cell resistance. These provide a theoretical basis for the combination of chemotherapy and immunotherapy. In a phase II trial, CRC patients were administered subcutaneously granulocyte macrophage colony-stimulating factor and low-dose interleukin-2, following gemcitabine + FOLFOX-4 (oxaliplatin, fluorouracil, and folinic acid) polychemotherapy. At a median follow up of 12.5 months, the ORR and DCR were as high as 68.9 and 96.5%, respectively. Analysis of peripheral blood mononuclear cells (PBMCs) in 20 patients showed that immune response to colon carcinoma antigen increased and suppressive regulatory T lymphocytes (CD4+CD25T-reg+) decreased significantly (102). Subsequent multicenter phase II and phase III clinical trials were conducted to further assess the combination (GOLFIG) in mCRC patients. In the phase II trial (GOLFIG-1 trial), including 46 mCRC patients who have had previous chemotherapy, RR and DCR were 56.5 and 91.3%, respectively, with a mean PFS of 12.3 months (103). In the phase III trial (GOLFIG-2), 124 mCRC patients were randomly assigned in a 1:1 ratio to receive the GOLFIG regimen or FOLFOX-4 regimen for the 1st line setting. Significant difference in RR (66.1% *vs*. 37·0%, P = 0.002), DCR, and PFS (9·23 *vs*. 5.70 months; P = 0·002) indicated that GOLFIG chemo-immunotherapy is markedly better than FOLFOX regimen for first-line treatment of mCRC (104). Caraglia et al. then retrospectively analyzed 179 mCRC patients in these two trials and followed them up for 15 years. Median PFS and OS were 15.28 (95% CI: 10.36–20.20) and 24.6 (95% CI: 19.07–30.14) months, respectively, To note, 14 patients survived for 10 years without disease progression (105). In their latest investigation of the GOLFIG-2 trial, patients in the GOLFIG group tend to achieve longer OS and PFS than those in the FOLFOX group (HR = 0.69, P = 0.06; HR = 0.58, p = 0.006).Their analysis also confirmed that pretreated patients had significant antitumor response, with a mean PFS of 12.55 (95% CI: 7.19–17.9) and OS of 20.28 (95% CI: 14.4–26.13) months, respectively. The GOLFIG regimen may be a reliable therapeutic option for pre-treated mCRC patients.

The above sequential clinical trials initially demonstrated the efficacy of chemotherapy combined with immunotherapy. The effect of chemotherapy on immune cells has been studied more and more. Roselli et al. analyzed mononuclear cell subsets from peripheral blood in mCRC patients (n = 23) before and during treatment with FOLFIRI plus bevacizumab. Despite differences among patients, most patients experienced small changes in the ratio of CD4(+) T cells to regulatory T cells (Treg) or small changes in Treg inhibitory activity during treatment. Tregs in responders to the chemotherapy was significantly decreased during therapy *vs*. pre-therapy compared with non-responders (106). In the same way, Scurr et al. observed a reduction in the percentage and absolute number of Treg in peripheral blood-derived lymphocytes from cyclophosphamide-treated mCRC patients. Cyclophosphamide significantly enhanced IFNγ+ tumor-specific T-cell responses and markedly delayed tumor growth in mCRC patients. [HR = 0.29; 95% CI, 0.12–0.69; P = 0.0047), compared with nonresponders and no-treatment controls (107). However, Dagenborg et al. reported that intratumoral T-cell densities was not associated with neoadjuvant chemotherapy therapy (NACT) before surgery in 45 mCRC patients. What is noteworthy is that intratumoral T-cell densities increased significantly in a short period of time aftertreatment, <9.5 weeks *vs* >9.5 weeks (medians 491, 236 cells/mm^2^, respectively; P <.0001).The results indicated that intratumoral T-cells may increase only for a short time after NACT administration, and the best combination of chemotherapy and immunotherapy should be further investigated (108). The relationship between chemotherapy and tumor PDL1 expression has also received more and more attention. Huang et al. found that expression of tumor PD-L1 and other immune-related genes were enhanced by decitabine (DAC)-induced DNA hypomethylation and intratumoral T cell infiltration increase *in vitro* and *in vivo* (109). Further, tumor samples from mCRC patients received Folfox regimen showed induction of PD-L1 expression and high CD8 T cell infiltration (110).

Other studies have shown that chemotherapy has a negative effect on immunotherapy. Bruni et al. reported that chemotherapy accelerates the aging of Vδ2pos T cells in CLM patients,which is non-classical lymphocytes possessing a wide range of anti-tumor activities (111). In 15 refractory mCRC patients treated with AMP-224 in combination with SBRT and low-dose cyclophosphamide, no one achieved objective response and three patients (20%) had stable disease. Patients did not benefit from the combination, with median PFS of 2.8 months (95%CI, 1.2–2.8 months) and OS of 6.0 months (95% CI, 2.8–9.6 months), respectively (112). Standard-of-care treatment seems to be harmful to early-stage CRC patients with high PD-L1 expression (HR = 4.95; CI, 1.10–22.35), suggesting that standard chemotherapy should not be used in stage II/III colorectal carcinoma patients with PD-L1 (high)/MSI/immune (high) (113).

### The Combination of Immunotherapy and Ablation

Ablation, as one of the established effective methods for resectable liver metastases from colorectal cancer, can also elicit tumor antigen-specific T cell responses and enhance the efficacy of immunotherapy. It can lead to tumor regression in untreated lesions, known as abscopal effect. This occurs because a variety of harmful molecules are released during ablation, including tumor-associated antigens, inflammatory cytokines, etc. In mouse models, we observed that incomplete radiofrequency ablation (iRFA) promotes tumor growth and impedes the efficacy of anti-PD-1 therapy. Mechanistically, more myeloid suppressor cells infiltrated into the local persistent inflammatory areas caused by iRFA, resulting in the inhibition of T cell function in tumors (114). Lemdani et al. demonstrated that TIL in metastatic lesion of patients and in mice model did not increase after RFA and RFA could not prevent recurrence. By adding systemic PD-1 blockade, immune deficiency in large secondary lesions can be reversed. In the situation of large lesions that do not respond to single RFA, the use of ICIs in metastatic MSS CRC may be reconsidered (115). Consistent with the above results, immunohistochemistry showed immune cells in metastatic lesions did not increase in six patients after RFA treatment, although induced immune responses and/or pre-existing T cell immunity against the specific targets was observed (116). Shi et al. reported that PD-L1-PD-1 axis plays a key role in inhibiting the antitumor immune responses induced by RFA. Not only T-cell infiltration, but also PD-L1 expression in primary human colorectal tumors increased after RFA treatment of liver metastases. Significantly enhanced T-cell immune responses, stronger antitumor immunity and prolonged survival were observed in mice model after the combined therapy of RFA and anti-PD-1 antibodies. This indicates the rationality and feasibility of ablation combined with immune checkpoint therapy for mCRC patients (117).

### The Combination of Immunotherapyand Radiotherapy

Another hot area of tumor immunotherapy is the use of monoclonal antibodies to deliver cytotoxic substances directly to the tumor site, known as radioimmunotherapy (RIT), which can increase the toxic dose of the tumor site and reduce the damage to the surrounding normal tissue. In a phase II study, 23 patients received RAT with radiolabeled anti-CEA antibodies after surgery for LM of CRC. At a median follow-up of 64 months, median OS and median DFS from initial hepatectomy for RAT patients was 68.0 months (95% CI, 46.0 months to infinity) and 18.0 months (95% CI, 11.0 to 31.0 months), with 5-year survival rate of 51.3%. Historical and contemporaneous controls without RAT were analyzed, adjuvant RAT seem to improve survival for CRC patients undergoing complete LM resection (118). Over a longer period of follow-up, Liersch et al. found 3- and 5-year survival rate of 68.4 and 42.1% for patients with RAT, compared with 36.8 and 15.8% for the controls (119). Some 13 patients are receiving the same type of RIT after complete resection of liver metastases (LM) from colorectal cancer. At a median follow-up of 127 months, median DFS and OS are 12 and 50 months, respectively (120). RIT targeting other antigen also showed safety and feasibility for 19 patients, with one patient of partial response, and 10 patients of stable disease (121). Studies have also evaluated the efficacy of RIT in combination with other treatments. The combination of cetuximab and RIT targeting CEA significantly reduced tumor growth and prolonged survival of mice than RIT monotherapy (122). Chen et al. demonstrated that RIT significantly increased PD-L1 expression on T cells. RIT plus PD-L1 blockade improved local tumor control, overall survival and avoid relapse, with expanded infiltration of CD8+ T cells (123).

## Other Types of Immunotherapy

Many experiments are also investigating the possibility of other molecules as future immunotherapies.

TGF-β is a kind of can influence a variety of cellular events and therefore has a dual role. On the one hand, this cytokine can block the ability of tumor cells to multiply by interfering with key molecules (CDK4, CDKI) in the cell cycle during the initial stages of cancer. On the other hand, TGF-β promotes tumor growth and metastasis as the tumors progress to an advanced stage (124). Many studies have demonstrated that blocking TGF-b signaling reduced metastasis in CRC and other solid tumors (125). Tauriello et al. discovered the significant role of TGF-β in the immune system for metastasis CRC. Increased TGFβ in the tumor microenvironment promoted immune evasion by decreasing T-cell infiltration and inhibiting acquisition of the TH1-effector phenotype. In the quadruple-mutant mice model bearing metastatic intestinal tumors with TGFβ-activated stroma, inhibition of TGFβ prevented metastasis by enhancing cytotoxic T-cell response against tumor cells, while the use of anti-PD1 drug drew finite efficacy. Furthermore, combination of TGFβ inhibitor and anti-PD1 drug had excellent effect in mice with severely hepatic metastases (126). Immunotherapies targeting TGFβ signaling and the combination with ICIs may therefore be potential and promising options for advanced CRC patients.

CC chemokines consist of 28 chemotactic cytokines crucial to all kinds of immune system cells, including CD4+ and CD8+ lymphocytes, dendritic cells, eosinophils, macrophages, monocytes, and NK cells. At the same time, they are essential in the development of tumors (127).

Halama et al. found that tumor-infiltrating lymphocytes delivering CCL5 are abundant in the invasive margin of hepatic metastatic samples from CRC patients, which instead promotes the growth and dissemination of tumor by polarizing macrophages to pro-tumoral phenotype *via* CCR5. Blocking CCR5 repolarized the macrophages to exert the anti-tumor efficiency *in vitro* organoid models, which was further confirmed in a phase I trial of CCR5 antagonist in refractory mCRC patients (128). Zhang et al. demonstrated that the lack of CCL5 inhibited tumor growth and metastasis by enhancing CD8+ T cells infiltration into tumor areas in CRC mouse models. Meanwhile, the absence of CCL5 could increase the PD-1 and PD-L1 expression and alleviate the resistance to ICIs in CRC mouse model. Clinical specimen from CRC patients also confirms the results (129). Same changes of immune-related molecules (CCR5, CCL5, PD1, PD-L1) in the microenvironment of hepatic metastases were also showed by Suarez-Carmona et al. By further analyzing two available cohorts, the data showed that patients with low gene expression of CCR5 in metastases had prolonged DFS (130). The study above suggests that targeting CCL5–CCL5 axis monotherapy or in combination with ICIs may be a possible therapeutic strategy for CRC and need to be tested in future trials.

Many studies have been looking at Toll-like receptors (TLRs) due to the ability of stimulating antitumor immunity by initiating innate and adaptive immune responses (131). 28 patients with metastatic solid tumors received a novel synthetic DNA-based toll-like receptor 9 (TLR9)-immunomodulator. In 15 patients completing the treatment cycle, six (40%) had stable disease (SD). NK cells, DCs and B cells were transiently increased, although there were no changes in the composition and activation status of various kinds of T cells (helper T cells, cytotoxic T cells, naive and memory T cells) (132). Sorski et al. found that the percentage of NK cells in marginating-hepatic (MH) cells was high in BALB/c mice, but the cytotoxicity was weak. However, TLR-9 agonist (CpG-C) treatment increased MH-NK cell numbers and activities in the mice model with mCRC, with increased maturation markers (NKp46, CD11b) and decreased the inhibitory NKG2D (133). In the murine colon metastatic cancer models, entolimod (TLR5 agonist) induces a large number of NK cells to migrate from blood and bone marrow to the liver (134). Then, we observed CD8(+) T-cell response following the activation of DCs depending on NK cells. Therefore, entolimod provoked tumor specific and persistent immune memory. TLR5 agonists can be used as efficient antitumor vaccine without the need to identify tumor-specific antigens. However, Zheng et al. found TLR ligands (TLR4, TLR5) released by nonvirulent tumor-targeting bacteria played a prominent part in tumor suppression in mouse models (135).

## Conclusion

**Figure 3:**
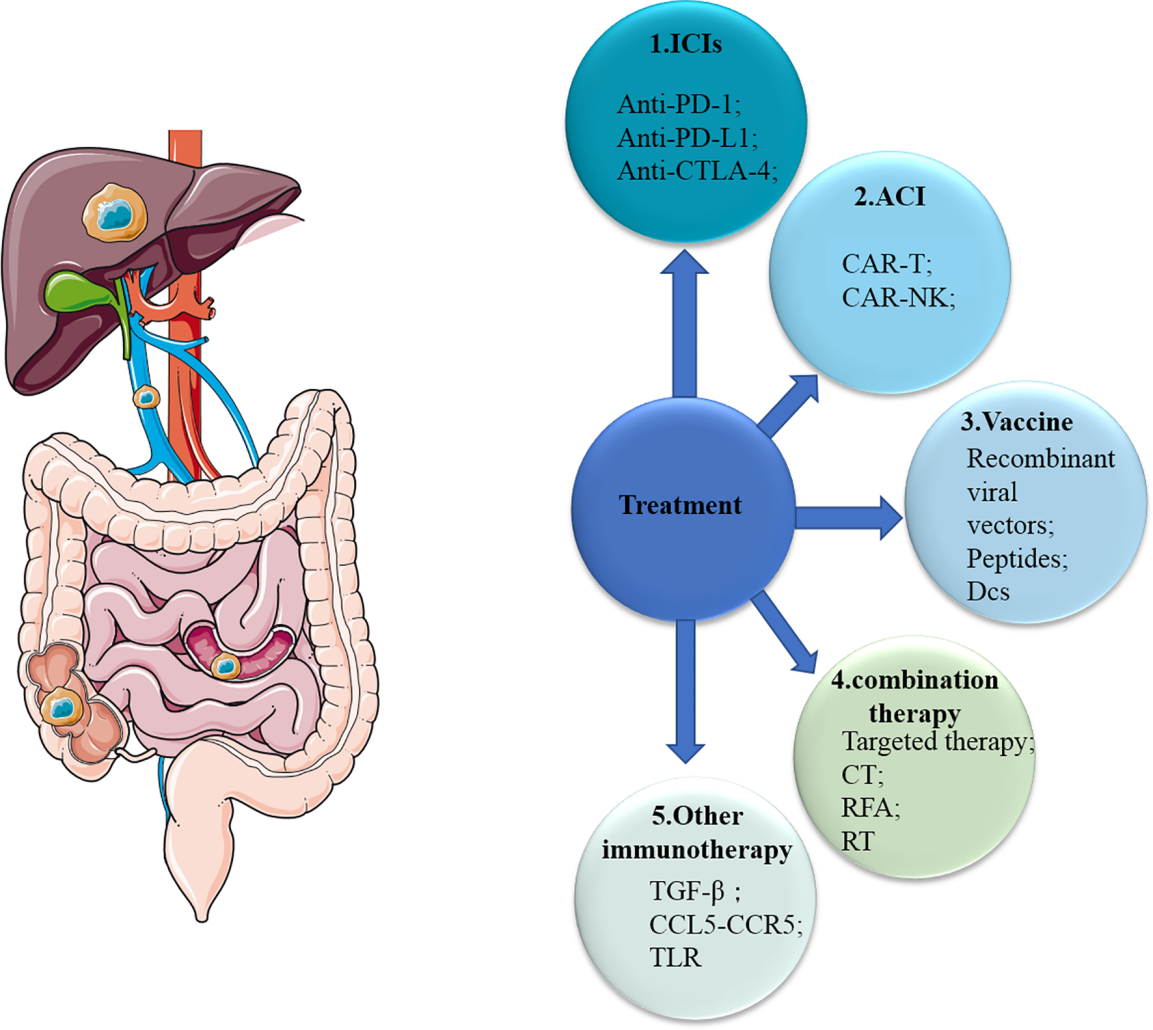
Overview of therapies for mCRC. mCRC, metastatic colorectal cancer; ICIs, immune checkpoint inhibitors; ACI, adoptive cellular immunotherapy; CT, chemotherapy; RFA, radiofrequency ablation; RT, radiotherapy; CTLA-4, cytotoxic T-lymphocyte-associated protein 4; PD-1, programmed cell death 1; PD-L1, PD-1 ligand; Treg, regulatory T cell; DC, dendritic cell; NK cell, natural killer cell; TLR, toll-like receptor.

In general, we are developing a variety of immunotherapies and achieved some successes in the field of immunotherapy, especially for mCRC patients with dMMR (**Figure 3**). However, for one thing, the response rate of these patients is still not high enough, and for another, there is no effective treatments including immunotherapy for other mCRC patients with MSS yet. First, ICIs are well researched, and there are already drugs approved for clinical use. Now, due to the relatively good advantages of molecular targeted therapies, more consideration should be given to the combination of ICIs and molecular targeted therapies (136). A small number of clinical trials have shown its potential with increased response rate and better prognosis, and more trials are underway and planned (**Table 3**). The results are worth waiting for. Secondly, few studies of ACT have been conducted in mCRC patients, although ACT has long been used in patients with hematological malignancies due to its excellent efficacy. We should explore more of its possibilities in mCRC patients, especially with regard to NK cell therapy. Thirdly, despite many studies, cancer vaccines have not made major breakthroughs in mCRC patients because of its limited role and possible safety issues. The cancer vaccines may be used more as an adjunct to other treatments to boost the immune response. Other molecules (TGF-β, CCL5, CCR5, toll receptor) found to affect the immune system are also promising. In addition, the development of High-Tech has made some progress in the application of nanotechnology in immunotherapy, mainly as a drug carrier (137, 138). In conclusion, although there are many challenges and problems, the possibilities of immunotherapy are endless.

**Table 3:** Ongoing or Future Clinical Trials of Immunotherapy for mCRC.

Study	Phase	Agent	Population	Status	Endpoint
**NCT03721653**	2	FOLFOXIRI + Bevacizumab + atezolizumab *vs* FOLFOXIRI + Bevacizumab	201 mCRC	-	The primary end point: DCR; The secondary endpoints: PFS, ORR, OS
**NCT03202758**	1/2	Durvalumab + Tremelimumab + FOLFOX	48 mCRC	-	The primary end point: safety
**NCT04072198**	2	Nivolumab + FOLFOXIRI/Bevacizumab	70 advanced CRC	RASm/BRAFm	The primary end point: ORR; The secondary endpoints: OS
**NCT03186326**	2	Avelumab versus a standard second-line chemotherapy plus a targeted agent according to tumor RAS status	132 mCRC	MSI/dMMR	The primary end point: median PFS; The secondary endpoints: ORR, OS, quality of life and toxicity
**NCT03827044**	3	Avelumab + 5-FU Based Chemotherapy	402 stage 3 CRC	MSI-High or POLE Mutant	The primary end point: DFS
**NCT04062721**	1	Local Immunomodulation (TLR agonist and GM-CSF) + Radiofrequency Ablation	50 mCRC	unresectable	The primary endpoints: PFS rate at 12 months; The secondary endpoints: median PFS; response rate; OS
**NCT04513431**	1	Anti-CEA-CAR T	18 mCRC	-	The primary endpoints: adverse effects including cytokine storm response and any other adverse effects
**NCT03698461**	2	Atezolizumab versus Atezolizumab + Bevacizumab + FOLFOX	20 mCRC	-	
**NCT04030260**	2	Regorafenib + Nivolumab + Radiotherapy	43 mCRC	pMMR/MSS	The primary endpoints: PFS rate at 6 months; The secondary endpoints: objective response; DCR; OS
**NCT04599140**	1/2	SX-682 + Nivolumab	53 mCRC	RAS Mutated; MSS	The primary end point: safety; The secondary endpoints: ORR
**NCT03202758**	1/2	Durvalumab + Tremelimumab + FOLFOX	48 mCRC	-	The primary end point: PFS; The secondary endpoints: OS
**NCT02754856**	1	Durvalumab + Tremelimumab	26 mCRC	-	The primary end point: safety and feasibility; The secondary endpoints: RFS

OS, overall survival; PFS, progression-free survival; DFS, disease-free survival; RFS, recurrence-free survival; ORR, objective response rate; irORR, immune-related objective response rate; DCR, disease control rate; BSC, best supportive care; PD-1, programmed cell death 1; PD-L1, programmed cell death ligand-1; CTLA-4, cytotoxic T-lymphocyte-associated protein 4; 5-FU/LV, 5-flourouracil, leucovorin; FOLFOX, 5-flourouracil, leucovorin, oxaliplatin; FOLFIRI, 5-flourouracil, leucovorin, irinotecan; mCRC, metastatic colorectal cancer; FOLFOXIRI, 5-flourouracil, leucovorin, oxaliplatin, irinotecan; MSI-(H), microsatellite instability-(high); MSS, microsatellite stable; TLR, toll-like receptor; CEA, carcinoembryonic antigen.

## Author Contributions

YD, WZ and LY were responsible for gathering information of the related research and designing the review. XD, DR and FW were responsible for language editing. XQ and JG have contributed to informatio0n interpretation, editing and critical revision of the manuscript. All authors contributed to the article and approved the submitted version.

## Conflict of Interest

The authors declare that the research was conducted in the absence of any commercial or financial relationships that could be construed as a potential conflict of interest.

## Abbreviations

mCRC, metastatic colorectal cancer; MSI/dMMR, microsatellite instability/mismatch-repair-deficiency; MSS/pMMR, microsatellite stability/mismatch-repair-proficiency; ICIs, immune checkpoint inhibitors; ACI, adoptive cellular immunotherapy; CT, chemotherapy; RFA, radiofrequency ablation; RT, radiotherapy; CTLA-4, cytotoxic T-lymphocyte-associated protein 4; PD-1, programmed cell death 1; PD-L1, PD-1 ligand; Treg, regulatory T cell; DC, dendritic cell; NK cell, natural killer cell; APC, antigen-presenting cell; TLR, toll-like receptor; OS, overall survival; PFS, progression-free survival; ORR, objective response rate; irORR, immune-related objective response rate; DCR, disease control rate; BSC, best supportive care; 5-FU, 5-flourouracil; 5-FU/LV, 5-flourouracil, leucovorin; FOLFOX, 5-flourouracil, leucovorin, oxaliplatin; FOLFIRI, 5-flourouracil, leucovorin, irinotecan; FOLFOXIRI, 5-flourouracil, leucovorin, oxaliplatin, irinotecan.
